# Sugar industry sponsorship of germ-free rodent studies linking sucrose to hyperlipidemia and cancer: An historical analysis of internal documents

**DOI:** 10.1371/journal.pbio.2003460

**Published:** 2017-11-21

**Authors:** Cristin E. Kearns, Dorie Apollonio, Stanton A. Glantz

**Affiliations:** 1 Philip R. Lee Institute for Health Policy Studies, University of California San Francisco, San Francisco, California, United States of America; 2 Department of Preventive and Restorative Dental Sciences, School of Dentistry, University of California San Francisco, San Francisco, California, United States of America; 3 Center for Tobacco Control Research and Education, University of California San Francisco, San Francisco, California, United States of America; 4 Department of Clinical Pharmacy, School of Pharmacy, University of California San Francisco, San Francisco, California, United States of America; 5 Helen Diller Family Comprehensive Cancer Center, University of California San Francisco, San Francisco, California, United States of America; 6 Cardiovascular Research Institute, University of California San Francisco, San Francisco, California, United States of America; 7 Department of Medicine; University of California San Francisco, San Francisco, California, United States of America

## Abstract

In 1965, the Sugar Research Foundation (SRF) secretly funded a review in the *New England Journal of Medicine* that discounted evidence linking sucrose consumption to blood lipid levels and hence coronary heart disease (CHD). SRF subsequently funded animal research to evaluate sucrose’s CHD risks. The objective of this study was to examine the planning, funding, and internal evaluation of an SRF-funded research project titled “Project 259: Dietary Carbohydrate and Blood Lipids in Germ-Free Rats,” led by Dr. W.F.R. Pover at the University of Birmingham, Birmingham, United Kingdom, between 1967 and 1971. A narrative case study method was used to assess SRF Project 259 from 1967 to 1971 based on sugar industry internal documents. Project 259 found a statistically significant decrease in serum triglycerides in germ-free rats fed a high sugar diet compared to conventional rats fed a basic PRM diet (a pelleted diet containing cereal meals, soybean meals, whitefish meal, and dried yeast, fortified with a balanced vitamin supplement and trace element mixture). The results suggested to SRF that gut microbiota have a causal role in carbohydrate-induced hypertriglyceridemia. A study comparing conventional rats fed a high-sugar diet to those fed a high-starch diet suggested that sucrose consumption might be associated with elevated levels of beta-glucuronidase, an enzyme previously associated with bladder cancer in humans. SRF terminated Project 259 without publishing the results. The sugar industry did not disclose evidence of harm from animal studies that would have (1) strengthened the case that the CHD risk of sucrose is greater than starch and (2) caused sucrose to be scrutinized as a potential carcinogen. The influence of the gut microbiota in the differential effects of sucrose and starch on blood lipids, as well as the influence of carbohydrate quality on beta-glucuronidase and cancer activity, deserve further scrutiny.

## Introduction

In 2017, whether fructose-containing sugars (e.g., sucrose) and starch have differential effects on blood lipids continues to be debated in the scientific literature [1–3]. Studies funded by the food and beverage industry or conducted by authors with food and beverage industry conflicts of interest have been critical of evidence indicating that fructose has unique metabolic effects, while those without such conflicts reach an opposite conclusion [4–6]. The seemingly intractable nature of this controversy may be rooted in more than 60 years of food and beverage industry manipulation of science. We previously reported, based on internal sugar industry documents, that the Sugar Research Foundation (SRF) secretly funded a 1967 review in the *New England Journal of Medicine* (*NEJM*) that discounted evidence linking sucrose consumption to coronary heart disease (CHD) [7]. Using the same methodology and internal document sources (see S1 Appendix), this paper presents data that suggest that in 1970, SRF withheld information from the public that the microbiome may be an important contributing factor to sucrose-induced hypertriglyceridemia and that sucrose consumption, compared to starch, might be associated with bladder cancer.

The Sugar Association, a United States sucrose industry trade association [8] (which has organizational ties to SRF, the International Sugar Research Foundation [ISRF], and ISRF’s successor, the World Sugar Research Organisation, based in London, UK [9]), has consistently denied [10–12] that sucrose has any metabolic effects related to chronic disease beyond its caloric effects. On January 5, 2016, the Sugar Association issued a press release [13] criticizing findings from a study published in *Cancer Research* [14] using multiple mouse models that suggested that dietary sugar induces increased tumor growth and metastasis when compared to a nonsugar starch diet. The Sugar Association stated that “no credible link between ingested sugars and cancer has been established.” In contrast, this paper provides empirical data suggesting that the sugar industry terminated funding of an animal study that was finding unfavorable results with respect to the association between dietary sugars and cancer, with possible translational importance to humans.

Our study contributes to a wider body of literature documenting industry manipulation of science. Industries seeking to influence regulation have a history of funding research resulting in industry-favorable interpretations of controversial evidence related to health effects of smoking [15,16], therapeutic effects of pharmaceutical drugs [17,18], the relationship between sugar-sweetened beverage consumption and weight gain or obesity [5], and the causes of climate change, [19] among other issues. The tobacco industry also has a long history of conducting research on the health effects of its products that is often decades ahead of the general scientific community and not publishing results that do not support its agenda [20–23]. This paper provides empirical data suggesting that the sugar industry has a similar history of conducting, but not publishing studies with results that are counter to its commercial interests.

### SRF launches Project 259

Based on its sponsorship of the 1967 *NEJM* review [24,25], SRF was aware of peer-reviewed published animal evidence suggesting a role of intestinal microbiota in the differential effects of sucrose and starch on blood lipids. The *NEJM* review [25] reported that starch-fed rats had significantly higher biliary excretion of bile acids [26] and lower serum cholesterol levels [27] than sucrose-fed rats. When the antibiotic sulfasuxidene was added to similar diets, the serum cholesterol level of the starch-fed rats rose, while in sucrose-fed rats, it did not [27], leading the *NEJM* review to report, “dietary influence on the intestinal [microbiota] was therefore suggested” [25].

In correspondence with *NEJM* review author D. Mark Hegsted in 1965, SRF Vice President of Research John Hickson posited that the differential effects of sucrose and starch on serum cholesterol might be explained by differences in the bacterial synthesis of thiamine in the intestine [28]. Hickson [28] referred to a 1964 paper, “Dietary Fats and Intestinal Thiamine Synthesis in Rats” [29], which summarized experimental evidence from animals indicating that the dietary requirement for thiamine was dependent on the type of carbohydrate consumed. The paper reported that rats fed a thiamine-deficient diet develop symptoms of a thiamine deficiency more rapidly when fed glucose or sucrose compared to potato starch and that thiamine deficiency was delayed with the administration of antibiotics. These results, according to the article, suggested that both starches and certain antibiotics encouraged the growth of thiamine-synthesizing gut bacteria, while sucrose did not. Hickson referred to the role of intestinal thiamine synthesis in the differential effects of sucrose and starch on blood lipids as:

Representative of a fact that has disturbed me for some time. The change from [sucrose] an essentially soluble [carbohydrate] to [raw starch] an essentially insoluble carbohydrate does provide a change in the [bacterial] synthesis [of thiamine in the intestine] and I have not been convinced that this factor has been eliminated [28].

In his correspondence with Hegsted, Hickson inquired about the possible role of intestinal thiamine synthesis in a then-recent clinical trial comparing the effect of carbohydrate quality on serum cholesterol levels conducted by Hegsted and colleagues [28]. In contrast to previous trials by other investigators, which had found differential effects of high-sucrose and high-starch diets on serum cholesterol, Hegsted and colleagues had found none. Hickson asked Hegsted whether these conflicting results might be related to differences in the thiamine status of experimental groups. It is not clear whether SRF communicated with Hegsted further about the role of gut microbiota and thiamine synthesis in the differential effects of sucrose and starch on blood lipids, but the industry continued to explore the topic. SRF launched Project 259 in 1968 [30] in an “attemp[t] to measure the nutritional effects of the [bacterial] organisms in the intestinal tract” when sucrose was consumed, compared to starch [31]. SRF explained Project 259’s rationale in an internal report:

It has been postulated that there might be a dietary significance in the indigestible residues of starch in the intestinal contents, which might account for the observed differences in the lipid carbohydrate-interactions between the simple sugars versus complex sugars [32].

SRF consulted with Professor Alastair Frazer, head of the Department of Clinical Biochemistry at the University of Birmingham, Birmingham, UK, to select the experimental model [33]. Despite the conclusion of the SRF-sponsored *NEJM* review that animal models had little value in evaluating sucrose’s CHD risks, SRF selected the germ-free rat for Project 259 (germ-free isolators to create and maintain germ-free laboratory animals had been developed in the 1940s [34]). In the 1960s, parallel studies with germ-free and conventional rats were considered a good model to examine the relationship between dietary factors, the gut microbiota, and blood lipids [29]. SRF chose W.F.R. Pover, a colleague of Frazer’s at the University of Birmingham, to lead the project. He was provided US$29,304 (US$187,583 in 2016 dollars) between June 1968 and September 1970 to conduct the study [31].

### Project 259 links sucrose consumption to cancer

**Fig 1:**
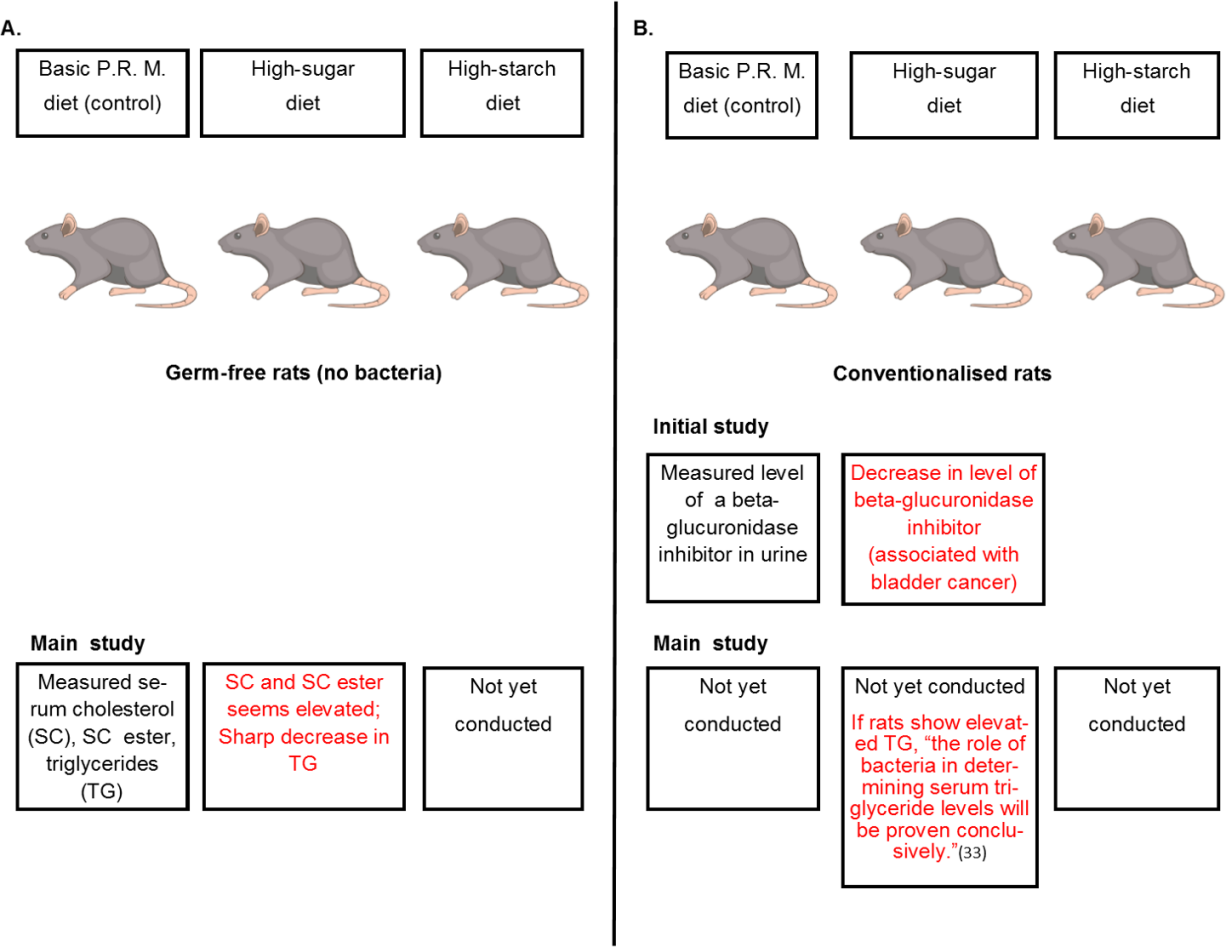
Experimental design for Project 259 and results reported to ISRF. (A) Project 259 was conducted using “germ-free” rats that were raised in isolators to limit their exposure to bacteria. The main study found rats fed a high-sugar diet showed a highly significant sharp decrease in triglycerides in the blood, compared to controls. (B) Project 259’s lead investigator, W.F.R. Pover, told ISRF that if the same rats showed an elevated triglyceride level after they were exposed to bacteria and fed the same high-sugar diet, “the role of bacteria in determining triglyceride levels will be proven conclusively [in rats]” [33]. ISRF terminated funding for the experiments before they could be completed. An initial preliminary study conducted before the main experiment found that rats fed a high-sugar diet had less of a beta-glucuronidase inhibitor in their urine than rats fed a basic PRM diet high in starch. Beta-glucuronidase is an enzyme, and high levels in the urine were known to be associated with bladder cancer in the 1960s. *Image of rat vector icon credit*: *Rvector/Shutterstock*.*com*. ISRF, International Sugar Research Foundation.

SRF, which became ISRF in July 1968, initially authorized 15 months of funding for Project 259 between June 1968 and September 1969 [35]. By ISRF’s June 1969 site visit to Pover’s lab, because of delays in receiving the equipment needed for the main experiment, Pover had conducted only initial studies with various rat strains and germ-free guinea pigs [32]. Pover’s initial experiments produced results that ISRF representatives found to be “of particular interest” (Fig 1B) [32]. According to its September 1969 Quadrennial Report of Research,

Among [Project 259’s] observations was … that the urine from rats on the basic diet contained an inhibitor of beta-glucorinidase activity in a quantity greater than that from sucrose-fed animals. *This is one of the first demonstrations of a biological difference between sucrose and starch fed rats* [emphasis added] [32].

The ISRF report did not include data from Project 259 and did not elaborate further on the experimental design of the studies or the significance of the finding that starch inhibited urinary beta-glucuronidase compared to sucrose.

Contemporaneous scientific publications, however, provide the context: elevated urinary beta-glucuronidase had been found to be positively associated with bladder cancer [36,37]. There was also some evidence that beta-glucuronidase activity was associated with atherosclerosis [38]. This incidental finding of Project 259 demonstrated to SRF that sucrose versus starch consumption caused different metabolic effects and suggested that sucrose, by stimulating urinary beta-glucuronidase, may have a role in the pathogenesis of bladder cancer.

### Project 259 links the microbiome to sucrose-induced hypertriglyceridemia

In August 1970, Pover reported to ISRF that the main germ-free experiment had achieved promising results (Fig 1):

There has been a sharp decrease in serum triglyceride of germfree rats fed a high sugar diet; there is no overlap with the serum triglyceride figures from rats fed the basic P.R.M. diet (a pelleted diet containing cereal meals, soybean meals, whitefish meal, and dried yeast, fortified with a balanced vitamin supplement and trace element mixture [39]) so this result is highly significant. We have yet to feed the starch diet. Both serum cholesterol and serum cholesterol ester values for germfree rats on sugar diet seem elevated; this result is not as clearcut as the triglyceride result and must await statistical evaluation before we can be sure.

The role of bacteria in determining serum triglyceride levels will be proven conclusively if we can demonstrate that these same rats, when conven[t]ionalised, show elevated serum T.G.s [triglycerides] when fed a sugar diet. This will take about 18 weeks [33].

ISRF had previously authorized an extension of Project 259’s funding to September 1970, which was 3 months short of the time Pover needed to conclude the experiment [33]. The language in Pover’s report suggests he was confident that Project 259, taken to completion, would confirm the causal role of gut microbiota in the differential effects of sucrose and starch on serum triglycerides in rats.

### ISRF terminates Project 259 funding

On September 10, 1970, as part of a strategic assessment of industry research conducted during the transition from SRF to ISRF, Hickson reported to industry executives on the contribution of SRF’s research projects to “elicit useful and significant information” to the sugar industry [33]. Hickson described the value of Project 259 as “nil” [33]. After supporting the project for 27 months, ISRF did not approve the additional 12 weeks of funding needed to complete the study. Knowing that additional funding was not forthcoming from ISRF, according to ISRF’s 1969–1970 Annual Report of Research, “Dr Pover ha[d] expressed hopes [to ISRF] of obtaining continuing support from other sources” [33]. No published papers were listed for Project 259 in the ISRF publication, *Sugar Research 1943–1972* [30]. We could not identify any published results.

A March 1974 ISRF report includes its internal interpretation of Project 259’s results:

Observations showed significant increase in serum triglyceride level with rats having conventional [microbiota] on sucrose diets, whereas a decreasing effect was noted with germ-free rats, suggesting the triglycerides were formed from fatty acids produced in the small intestine by the fermentation of sucrose [30].

ISRF’s summary of Project 259 confirms that the sugar industry interpreted the results as indicating that intestinal bacteria had a role in sucrose-induced hypertriglyceridemia in rats.

### Implications

Despite limited available detail about Project 259’s study design, it appears that ISRF did not terminate funding because of concerns about the quality of the study. ISRF’s June 1969 site visit to Pover’s laboratory suggests that ISRF was informed of and perhaps had input into the study design. Indeed, ISRF’s internal reports show how ISRF interpreted the results: biological differences between sucrose- and starch-fed rats were demonstrated [32] and a mechanism by which sucrose caused triglyceride formation was suggested [30].

Based on ISRF’s interpretation of preliminary results, extending Project 259’s funding would have been unfavorable to the sugar industry’s commercial interests. In the 1960s, scientists disagreed about whether sucrose was hypertriglyceridemic relative to starch [40]. Project 259’s preliminary results, if confirmed upon project completion and subsequently published, would have supported the argument that sucrose was hypertriglyceridemic. ISRF’s decision to terminate Project 259’s funding was also consistent with SRF’s earlier efforts to cast doubt on the CHD risks of sucrose [41].

In addition, publication of results suggesting an association between sucrose consumption and bladder cancer would likely have had further adverse regulatory implications to the sugar industry. As of 1958, the US Food Additives Amendment stated that any food found to cause cancer when ingested by animals was grounds for removal from the Food and Drug Administration’s list of foods generally recognized as safe (GRAS) [42]. Project 259’s September 1969 finding indicated that the urine of rats fed a high-sucrose versus a high-starch diet contained higher levels of beta-glucuronidase, an enzyme that had been previously associated with bladder cancer in rats [36,37]. Had ISRF disclosed Project 259’s findings, it is likely that sucrose would have received scrutiny as a potential carcinogen. This possibility seems particularly likely because in October 1969, the FDA removed cyclamates—artificial sweeteners that had captured significant market share from sucrose—from the GRAS list based on evidence that rats fed high levels of cyclamates developed bladder tumors [43].

It is not clear why the results of Project 259 were never published. One possibility is that Pover was unable to locate timely funding from other sources to allow the experiments to be completed. Another possibility is that the project was completed, but the results were unpublishable. Regardless of what actually happened, these events would have occurred after ISRF’s decision to terminate funding and would, therefore, not have informed ISRF’s decision to terminate funding shortly before the data collection phase of the project was complete. Because ISRF knew that Pover did not have a new funder lined up [33], it is plausible to believe that ISRF thought that terminating Project 259’s funding would prevent completion of the project and publication of results that were potentially damaging to the sugar industry.

Our analysis also identifies several lines of inquiry active in the 1960s about the health effects of added sugars, which may warrant further investigation today. One is the role of thiamine deficiency in differential effects of sucrose versus starch on blood lipids. Thiamin is a water-soluble vitamin that acts as a coenzyme in reactions critical to normal carbohydrate metabolism obtained either from dietary sources or from microbial synthesis in the large intestine [44]. Although investigators were aware in the 1960s that carbohydrate quality influences intestinal thiamine synthesis and subsequent blood lipid levels, the contribution of bacterially synthesized thiamin to health remains unclear [45].

Another line of inquiry active in the 1960s that may deserve attention is the relationship between carbohydrate quality, urinary beta-glucuronidase, and bladder cancer. Since the 1960s, urinary beta-glucuronidase activity has also been associated with renal disorders [46], urinary tract infections [47,48], and renal transplant rejection [49]. Beta-glucuronidase is an enzyme produced in most human tissues that cleaves glucuronic acid from substrates, making them less water soluble and inhibiting excretion [50]. Substrates include drugs, dietary factors, toxins, and steroid hormones, among others. While it has been hypothesized that factors inhibiting serum beta-glucuronidase lower cancer risk [50], little is currently known about dietary correlates of beta-glucuronidase activity. The results from Project 259 suggest that carbohydrate quality may modulate urinary beta-glucuronidase.

## Supporting information

S1 Appendix Methods. (PDF)

## Abbreviations

CHD: coronary heart disease
GRAS: generally recognized as safe
ISRF: International Sugar Research Foundation
NEJM: *New England Journal of Medicine*
SRF: Sugar Research Foundation
