# Supplementary material for: Sugar industry sponsorship of germ-free rodent studies linking sucrose to hyperlipidemia and cancer: An historical analysis of internal documents
Source: PLoS Biol. 2017 Nov 21;15(11):e2003460. doi: 10.1371/journal.pbio.2003460 (PMC5697802; doi:10.1371/journal.pbio.2003460)
Supplement: S1 Appendix — (PDF) [file pbio.2003460.s001.pdf]

Sugar Industry Sponsorship of Germ-Free Rodent Studies Linking Sucrose to Hyperlipidemia and Cancer: An Historical Analysis of Internal Documents

Cristin E. Kearns, Dorie Apollonio, Stanton Glantz

SUPPORTING INFORMATION

S1 Appendix: Methods

This study examines the planning, funding, and internal evaluation of a Sugar Research Foundation (SRF)-funded research project titled "Project 259: Dietary Carbohydrate and Blood Lipids in Germ-Free Rats" led by Dr. W.F.R. Pover at the University of Birmingham, Birmingham, UK between 1967 and 1971. Qualitative methods guided this study because it is a form of inquiry that analyzes information conveyed through language and behavior in natural settings [1]. Data sources included: 1) organizational documents produced by SRF and its successor, the International Sugar Research Foundation (ISRF) including meeting minutes, research reports, press releases, and correspondence; 2) personal documents produced by scientists who consulted and/or received research funding from SRF/ISRF; 3) scientific publications referenced in organizational or personal documents; 4) scientific publications funded by SRF/ISRF; and 5) contemporaneous scientific publications that provided context to research topics and design.

We searched for SRF/ISRF organizational documents and publications using the Google search engine, and in the library catalog of over 10,000 worldwide libraries contained in the OCLC WorldCat network. Search terms included "Sugar Research Foundation," "International Sugar Research Foundation," and "archive." We searched for personal documents related to SRF/ISRF using the Google search engine, the OCLC WorldCat network, and in ArchiveGRID,

an online search engine with access to information about primary source materials in over 1,000 archival institutions.

In consultation with Archival Reference and Operations Specialist staff at the University of Illinois Archives, the first author identified organizational and personal documents related to SRF and ISRF, including correspondence with sugar industry executives, meeting minutes, and other reports in the Roger Adams papers in the University of Illinois Archives (319 documents totaling 1,551 pages) in 2012 [2]. Roger Adams, Emeritus Professor of Organic Chemistry, served on the SRF and ISRF Scientific Advisory Board [3] from 1959 until his death in 1971 [4,5]. The pages were initially photocopied, then scanned and made searchable using optical character recognition technology. Also in 2012, the first author visited The Francis A. Countway Library of Medicine in Boston, MA to visually inspect the D. Mark Hegsted papers [6]. Hegsted was a Professor of Nutrition at the Harvard School of Public Health and co-director of SRF's first coronary heart disease (CHD) research project [7] which resulted in a secretly-funded 1967 review in the *New England Journal of Medicine* that discounted evidence linking sucrose consumption to CHD [8,9]. Portions of the collection were selected for viewing by consulting the collection's finding aid [10] and a Reference Archivist. Twenty-seven organizational and personal documents were identified as related to SRF/ISRF (totaling 31 pages) [11]. Digital photographs were taken of the documents.

An electronic search for scientific publications related to SRF Project 259 was conducted in the following databases in September, 2016: PubMed (1946-2017), Embase (1947-2017), and Web of Science (1952-2017), using search terms ("Pover W" OR "Pover WF" OR "Pover WFR") and found no publications. A second search was conducted in January 2017 and found no publications. Contemporaneous scientific publications that provided context to research topics

were found in PubMed using search terms ("germ-free" OR "beta-glucuronidase," OR "beta-glucoronidase") for articles published between 1950 and 1980.

Prior to analysis, all organizational and personal documents were chronologically organized to provide an overview of how Project 259 progressed. All textual documents collected from all sources were read and evaluated for relevance to Project 259. The first author conducted an initial reading and note-taking of the data. Analysis occurred across all textual documents guided by a thematic analysis. Emerging themes relevant to CHD research were developed by reviewing the documents repeatedly and considering possible meanings and how these fit with developing themes [12]. Findings were assembled chronologically into a narrative case study.

The organizational and personal documents used in this research provide a narrow window into the activities of one sugar industry trade association; therefore, it is difficult to validate that the documents gathered are representative of the entirety of SRF/ISRF internal materials related to Project 259 or that the proper weight was given to each data source. There is evidence that ISRF terminated its funding of Project 259, but no direct evidence that this prohibited the researchers from concluding the project. We could not interview key actors involved in this historical episode because they have died.

REFERENCES

1. Lincoln YS, Guba EG. Naturalistic inquiry. Beverly Hills, Calif.: Sage Publications; 1985.

2. Roger Adams: An inventory of the Papers of Roger Adams at the University of Illinois Archives, 1889-1971. Papers of Roger Adams. Record Series Number 15/5/23. Urbana, Illinois:

University of Illinois Archives. Available from:
https://archives.library.illinois.edu/ead/ua/1505023/1505023f.html

3. Adams R. Letter to Ernest W. Greene, President of the Sugar Association (September 17). Papers of Roger Adams. Record Series Number 15/5/23. Urbana, Illinois: University of Illinois Archives; 1959.

4. Ross P. Letter to Roger Adams of Sugar Research Foundation Scientific Advisory Board (October 3) Papers of Roger Adams. Record Series Number 15/5/23: Urbana, Illinois: University of Illinois Archives;1968.

5. Adams R. Letter to John L. Hickson, Vice President of International Sugar Research Foundation (February 26). Papers of Roger Adams. Record Series Number 15/5/23. Urbana, Illinois: University of Illinois Archives; 1971.

6. D. Mark Hegsted Papers, 1952-1999 (inclusive), 1960-1978 (bulk). H MS c54, Boston, Mass: Harvard Medical Library, Francis A. Countway Library of Medicine.

7. Cheek DW. Sugar research, 1943-1972. Bethesda: International Sugar Research Foundation; 1974.

8. McGandy RB, Hegsted DM, Stare FJ. Dietary fats, carbohydrates and atherosclerotic vascular disease. New Engl J Med. 1967;277(4):186.

9. McGandy RB, Hegsted DM, Stare FJ. Dietary fats, carbohydrates and atherosclerotic vascular disease. New Engl J Med. 1967;277(5):242-7.

10. Hegsted, D. Mark (David Mark), 1914-2009. Papers, 1952-1999 (inclusive), 1960-1978 (bulk): Finding aid. H MS c54. Boston, Mass: Harvard Medical Library, Francis A. Countway Library of Medicine. Available from: http://oasis.lib.harvard.edu/oasis/deliver/~med00138.

11. D. Mark Hegsted Papers 1952-1991. Series II. General Correspondence, 1952-1992. D Mark Hegsted Papers, 1952-1999 (inclusive), 1960-1978 (bulk) H MS c54. Boston, Mass: Harvard Medical Library, Francis A. Countway Library of Medicine.

12. Gibbs G. Analyzing qualitative data. London: Sage; 2007.
